# Prevalence of synonymous mutations in m^6^A modification sites in human cancers

**DOI:** 10.1016/j.gendis.2024.101373

**Published:** 2024-07-06

**Authors:** Richard Chen, Lu Yang, Li Han, Zunsong Hu, Zhaohui Gu, Xiaolan Deng

**Affiliations:** a Department of Systems Biology, Beckman Research Institute of City of Hope, Monrovia, CA 91016, USA; b Department of Computational and Quantitative Medicine, Beckman Research Institute of City of Hope, Monrovia, CA 91016, USA; c South Pasadena High School, 1401 Fremont Ave, South Pasadena, CA 91030, USA; d Center for RNA Biology and Therapeutics, City of Hope, Duarte, CA 91010, USA; e School of Pharmacy, China Medical University, Shenyang, Liaoning 110001, China

As one amino acid can often be encoded by multiple codons, the genetic code is redundant, which accounts for synonymous mutations in protein-coding regions.^1^ Since synonymous mutations do not cause any alterations in amino acid sequence, it was previously believed that they do not change the structure and function of the proteins and thus are functionally silent.^1^ However, evidence is emerging that synonymous mutations can affect messenger RNA (mRNA) stability, splicing and translation, and thereby influencing protein biogenesis.^1^ Nevertheless, the detailed mechanisms underlying the impacts of synonymous mutations on mRNA metabolism and protein biogenesis remain elusive. *N*^6^-methyladenosine (m^6^A), the most prevalent internal modification in eukaryotic mRNA, has been shown to play important roles in gene regulation in both normal and disease cells, through post-transcriptional regulation of mRNA stability, splicing and translation.^2^ Therefore, we assumed that if synonymous mutations occur in RNA m^6^A modification sites, synonymous mutations could change mRNA m^6^A modifications, which in turn would affect mRNA stability, splicing and translation. Indeed, through overlapping synonymous mutations in the classic m^6^A motif DR**A**CH (*D* = A, G or U; R = G/A; H = A, C or U) sites in human cancers listed in the COSMIC (the Catalogue of Somatic Mutations in Cancer) database (https://cancer.sanger.ac.uk)^3^ with the detected m^6^A peaks listed in the RMBase database,^4^ we found that around 4500 synonymous mutations in over 3000 genes occurred at the “A” site of the classic m^6^A motifs DR**A**CH with detected m^6^A peaks. Thus, our data reveals the prevalence of overlapping between synonymous mutations and m^6^A sites in genes in human cancers, suggesting that synonymous mutations-mediated m^6^A modification changes may contribute to the changes in the expression of the target genes in human cancers.

Briefly, we searched for all the synonymous mutations that occurred at the “A” position of the classic m^6^A motifs DR**A**CH from the COSMIC database, and then overlapped them with the detected m^6^A peaks listed in the RMBase database. We identified a total of 4487 synonymous mutations (see Table S1) occurred at the “A” position of the classic m^6^A motifs DR**A**CH that is associated with detected m^6^A peaks, with “GA**A**CA”, “GG**A**CA” and “AA**A**CA” as the top three m^6^A motifs with such synonymous mutations (see Fig. 1A). These 4487 synonymous mutations occurred in a total of 3132 genes, with 26% of them (816 genes) containing at least 2 synonymous mutations (Fig. 1B). Such mutations occurred in various types of human cancers, with the highest frequency in colorectal cancer, endometrial carcinoma, and gastric cancer, all of which have more than 480 such mutations detected (see Fig. 1C). Notably, these cancers are not classified as the cancer types with hyper-tumor mutational burden,^5^ suggesting that the high frequency of such synonymous mutations in these cancer types is not simply due to the hyper-tumor mutational burden. We then conducted gene ontology (GO) Biological Process (BP) analysis and found that the genes with such synonymous mutations are significantly enriched in the major biological processes such as “regulation of primary metabolic process”, “regulation of RNA metabolic process”, “regulation of biological process”, “RNA biosynthetic process”, “cell cycle”, and so on (Fig. 1D). Our GO Molecular Function (MF) analysis revealed that the genes with such synonymous mutations are significantly enriched in “ion binding”, “small molecule binding”, “protein binding”, “DNA binding”, and so on (Fig. 1E). We also conducted Reactome (REAC) pathway analysis, and found that the genes with such synonymous mutations are significantly enriched in “Gene expression (Transcription)”, “RNA Polymerase II Transcription”, “Generic Transcription Pathway”, “RHO GTPase cycle”, and so on (Fig. 1F).

**Figure 1:**
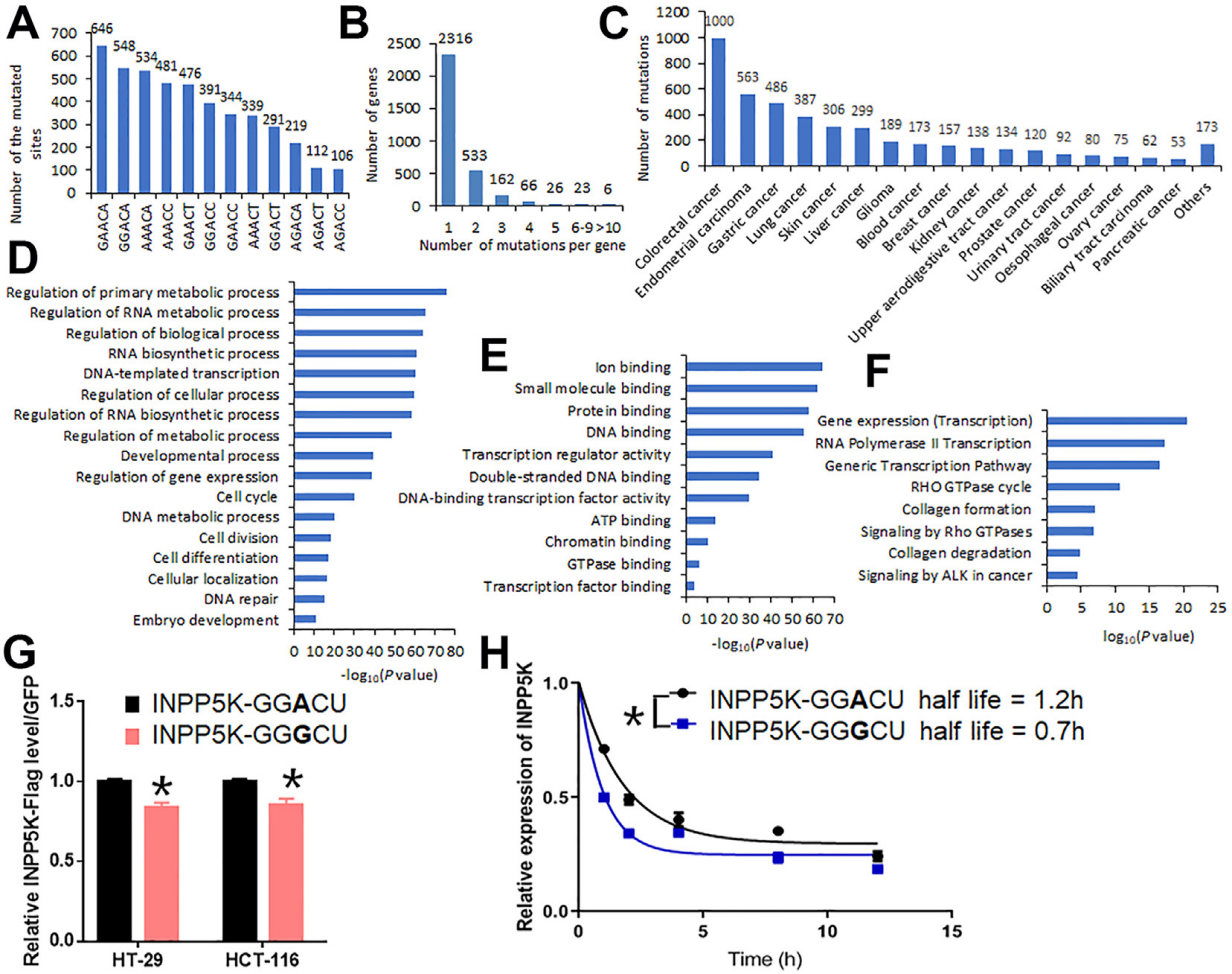
Prevalence of synonymous mutations occurring at the “A” site of the classic m^6^A motifs DRACH with detected m^6^A peaks, and their potential impacts. **(A)** The numbers of synonymous mutations occurring at the “A” site of different m^6^A motifs with detected m^6^A peaks. **(B)** The numbers of individual genes that have different numbers of such synonymous mutations. **(C)** The numbers of such synonymous mutations detected in different types of human cancers. Others, other types of cancers besides the listed individual types of cancers. **(D**–**F)** GO analysis of the genes with such synonymous mutations enriched in the specific Biological Processes (BP; D), Molecular Functions (MF; E), and Reactome pathways (REAC; F). **(G)** Comparison of mRNA levels between forced expressed wildtype *INPP5K* (INPP5K-GG**A**CU) and mutant *INPP5K* with a synonymous mutation (INPP5K-GG**A**CU) in HT-29 and HCT-116 cell lines. Their mRNA levels were normalized with GFP mRNA levels, and then the wildtype *INPP5K* mRNA level was set to 1. **(H)** Comparison of RNA stability between forced expressed wildtype *INPP5K* (INPP5K-GG**A**CU) and mutant *INPP5K* with a synonymous mutation (INPP5K-GG**A**CU) in HT-29 cells. ∗, *P* < 0.05. *t*-test.

If we excluded all the mutations that are also known to be associated with Single Nucleotide Polymorphisms (SNPs), there would be 4286 synonymous mutations left. Of them, 1524 synonymous mutations have been reported in the literature collected in the PubMed, and they are more reliable synonymous mutations. Among these 1524 more reliable synonymous mutations, “GA**A**CA”, “GG**A**CA” and “AA**A**CA” remain the top three m^6^A motifs with such synonymous mutations (see Fig. S1A). These 1524 synonymous mutations occurred in 1276 individual genes, with ∼39% of the genes containing at least 2 synonymous mutations (Fig. S1B). Such genes are significantly enriched in “Regulation of primary metabolic process”, “Transcription by RNA polymerase II”, “Regulation of biological process”, “RNA biosynthetic process”, “DNA binding”, “Protein binding”, “Ion binding”, “Gene expression (Transcription)”, “RNA Polymerase II Transcription”, “Generic Transcription Pathway”, and so on (see Fig. S1C,S1E). There are a total of 201 SNP-associated synonymous mutations with PubMed reports. Notably, “GG**A**CC”, “AA**A**CA” and “GG**A**CA” are the top three m^6^A motifs with such synonymous mutations (see Fig. S1F). These 201 SNP-associated synonymous mutations occurred in 194 individual genes, with 7 genes containing 2 synonymous mutations and the remaining 187 genes with only one synonymous mutation. Such SNP-associated mutations occurred in various types of human cancers, with the highest frequency in blood cancer, breast cancer and colorectal cancer, all of which have at least 27 SNP-associated synonymous mutations detected (Fig. S1G).

As m^6^A can post-transcriptionally regulate mRNA stability, splicing and translation,^2^ the aforementioned synonymous mutations could cause the loss of the associated m^6^A modification and thereby affect the fate of the target mRNAs. To test this, we randomly selected a synonymous mutation (c.1092 A  >  G) occurred in a classic m^6^A motif “GG**A**CU” in gene *INPP5K* (Inositol polyphosphate-5-phosphatase K) in colorectal cancer (see Table S1) as a representative for our verification study. *INPP5K* has been reported to be a tumor-suppressor gene and its low expression level is associated with poor prognosis in liver cancer patients. We established lentiviral constructs containing the FLAG-tagged coding region (CDS) of either wild type (with GG**A**CU) or mutated (c.1092 A  >  G; with GG**G**CU) *INPP5K*, and then transduced them into two colorectal cancer cell lines, HCT-116 and HT-29; both cell lines contain the endogenous wild type *INPP5K* (see Fig. S2A). As the lentiviral vector can also simultaneously express GFP, we sorted out GFP positive (GFP^+^) cells after the viral transduction for further analysis (see Fig. S2B,S2C). As shown in Figure 1G, forced expressed *INPP5K* with the synonymous mutation (c.1092 A  >  G) has a significantly lower mRNA abundance (*p* < 0.05) in both HCT-116 and HT-29 cell lines. We then conducted mRNA stability assay and confirmed that this mutation could significantly shorten the half-life of *INPP5K* mRNA (see Fig. 1H).

In summary, here we show for the first time that there are thousands of synonymous mutations that occurred at the “A” position of the classic m^6^A motifs DR**A**CH with m^6^A peaks in human cancers. Such mutations would not change the sequence of amino acids, but instead they would lose the associated m^6^A modification and thereby affect the fate of the target mRNAs, leading to dysregulated RNA splicing, stability and/or translation. Some of such synonymous mutations may even serve as the driver mutations in cancer initiation and progression. In addition, besides human cancers, other types of human diseases certainly also contain such synonymous mutations that affect m^6^A modification and may play important roles in disease development. In the clinical diagnosis practice, many disease patients have been found to have synonymous mutations, but such mutations have often been omitted from the analysis and reports due to no amino acids changed and thus are not considered as pathological mutations. Our results reveal a new mechanism underlying the pathological roles of synonymous mutations in human diseases, in which synonymous mutations alter the relevant m^6^A modifications and thereby affect target mRNA fate and gene expression, subsequently contributing to the development of human diseases, including cancers. Therefore, it is important and interesting to systematically study such synonymous mutations in human cancers and other types of diseases in the future, which will not only lead to the identification of diagnosis and prognosis biomarkers, but also may lead to the development of improved novel therapeutics.

## Author contributions

R.C. and X.D. conceived and designed the project. Z.G. and X.D. supervised the experiments and/or data analysis done by their groups. R.C., L.Y., L.H., Z.H., and X.D. conducted experiments and/or data analysis. R.C. and X.D. wrote the manuscript and all the authors provided feedback.

## Conflict of interests

The authors declare no competing interests.

## Funding

This work was supported in part by the Eugene and Ruth Roberts Summer Student Academy Program of City of Hope (to R.C.).
