# Supplementary material for: Prevalence of synonymous mutations in m6A modification sites in human cancers
Source: Genes Dis. 2024 Jul 6;12(1):101373. doi: 10.1016/j.gendis.2024.101373 (PMC11549976; doi:10.1016/j.gendis.2024.101373)
Supplement: Multimedia component 1 [file mmc1.docx]

**Supplemental Materials and Methods**

**Identification of synonymous mutations occurring at the “A” site of the classic m^6^A motifs DRACH associated with m^6^A peaks**

Mutations in the DRACH (D=A, G or U; R = G/A; H=A, C or U) motif were scanned in the the COSMIC (the Catalogue of Somatic Mutations in Cancer) database (https://cancer.sanger.ac.uk)[^3^](#_ENREF_3) (V98) database and cross-examined with human m^6^A peaks from the RMBase database[^4^](#_ENREF_4) (V3.0) using the following steps: (i) First, mutation records were selected where the reference (REF) allele is 'A'; (ii) Second, records were further filtered to include only mutations where the REF=A allele occurs in the 'A' position of the DRACH motif; (iii) Third, the mutation locations were checked for overlap with the list of m6A peaks from RMBase V3.0; (iv) Fourth, only synonymous mutations were selected, and a combination of the "Gene, Seqnames, POS, REF, ALT, LEGACY_MUTATION_ID" column identifiers was used to ensure unique mutation records (resulting in 4,487 unique synonymous mutations); and (v) Fifth, rsID annotations were added to the result table based on the mutation locus overlapping with the dbSNP151 database.

**Pathway analysis**

The pathway analysis was conducted using g:Profiler (https://biit.cs.ut.ee/gprofiler/gost, accessed on: 2/22/2024). Gene lists were input into the query dialog and the ‘Run query’ button was clicked to perform the analysis, selecting Homo sapiens as the organism. In the advanced options, 'Only annotated genes' was chosen for the statistical domain scope, 'g:SCS threshold' was selected for the significance threshold method, and '0.05' was set as the significance threshold level. Numeric IDs were treated as ENTREZGENE_ACC. The pathway databases and their respective versions utilized were as follows: GO BP and MF: BioMart, classes released on 2024-01-17; REAC: BioMart, classes dated 2024-01-25.

**Cell culture**

The human colorectal carcinoma cell line HCT-116 and HT-29 were cultured in McCoy's 5A medium supplemented with 10% FBS (Invitrogen). HEK-293T cells were grown in DMEM medium (Invitrogen) containing 10% FBS. All media were supplemented with Penicillin-Streptomycin and Plasmocin prophylactic to prevent potential contamination. The identities of all cell lines were authenticated by short tandem repeat (STR) analysis. All cell lines were routinely tested for mycoplasma contamination by Mycoplasma PCR Detection Kit (G238, Applied Biological Materials Inc.).

**Plasmid construction, Lentivirus preparation and infection**

The wild type *INPP5K*-GGACU were PCR-amplified using cDNA as templates, and then ligated into pCDH to generate pCDH-Flag-tagged INPP5K-GGACU with In-Fusion HD Cloning Plus Kits (638909, Takara Bio). The pCDH-Flag-tagged INPP5K-GGGCU were constructed based on pCDH-FLAG-tagged INPP5K-GGACU. All the primers used for In-fusion cloning are listed in Table S2. All plasmids constructed in this study were extracted with QIAprep Spin Miniprep Kit (27106, Qiagen) and then confirmed by Sanger sequencing (Eton Bioscience Inc.).

Lentivirus particles for overexpression INPP5K-GGACU and INPP5K-GGGCU plasmids were produced in HEK-293T cells with the 2nd-generation package system. Briefly, 2.25 μg psPAX2, 0.75 μg pMD2.G, and 3 μg target plasmids were co-transfected into HEK-293T cells with X-tremeGENE™ HP DNA Transfection Reagent (6366546001, Sigma-Aldrich) in 60mm cell-culture dishes. Lentivirus particles-containing supernatant was harvested at 48 and 72 hours after transfection and centrifuged for 30min at 3000g at 4°C. For infection, the viral supernatant was directly added into cells in the presence of 4 ug/ml polybrene (H9268, Sigma-Aldrich). After 48-hour infection, cells were monitored for GFP expression by flow cytometry, with approximately 15% infection and then selected with 2 μg/mL puromycin.

**DNA Isolation and Genotyping by DNA Sequencing**

Genomic DNA was extracted from HCT-116 and HT-29 cells using the QIAamp DNA Mini Kit (56304, Qiagen) following the manufacturer’s instructions. Multiple PCR was performed with 10-100 ng of genomic DNA to amplify INPP5K, which contained c.1092A, using CloneAmp™ HiFi PCR Premix (639298, TAKARA) following the manufacturer’s instructions. The PCR products were purified with DNA Clean & Concentrator-25 (D4033, ZYMO RESEARCH) and then sequenced using the forward primer. All the primers used in PCR analysis are listed in Table S2.

**RNA Extraction and quantitative RT-PCR analysis**

Total RNA was isolated from cells using TRIzol reagent following the manufacturer’s instructions. Reverse transcriptase reaction was performed with QuantiTect Reverse Transcription kit (205314, QIAGEN) according to the manufacturer’s instructions. Quantitative real-time PCR (qPCR) was carried out with PowerUp™ SYBR™ Green Master Mix (A25741, Thermo Fisher). Target gene RNA expression levels were normalized by reference gene, β-actin. The qPCR primers used in this study are listed in Table S2.

**RNA Stability assay**

The cells with either INPP5K-GGACU or INPP5K-GGGCU overexpression were treated with Actinomycin D (A9415, Sigma-Aldrich) to assess RNA stability of INPP5K-GGACU and INPP5K-GGGCU transcripts. After incubation for indicated time, cells were harvested. Total RNA was extracted with TRIzol reagent for qPCR analysis. The half time of mRNA were calculated according to previously described. Since Actinomycin D treatment leads to transcription inhibition, the mRNA degradation rate k_decay_ was estimated by following equation:

In(C/C_0_) = -K_decay_t

C_0_ is the concentration of mRNA at time 0, t is the transcription inhibition time, and C is the mRNA concentration at the time t. To calculate the half-time (t1/2), indicating when 50% of mRNA has decayed (C/C_0_ = 50%/100% = 1/2), the equation can be:

In(1/2) = -K_decay_t_1/2_

from where:

t_1/2_ = In2/K_decay_
