# Supplementary material for: Prevalence of synonymous mutations in m6A modification sites in human cancers
Source: Genes Dis. 2024 Jul 6;12(1):101373. doi: 10.1016/j.gendis.2024.101373 (PMC11549976; doi:10.1016/j.gendis.2024.101373)
Supplement: Multimedia component 2 [file mmc2.docx]

**Supplementary Data**

**Supplemental Figures and Legends**


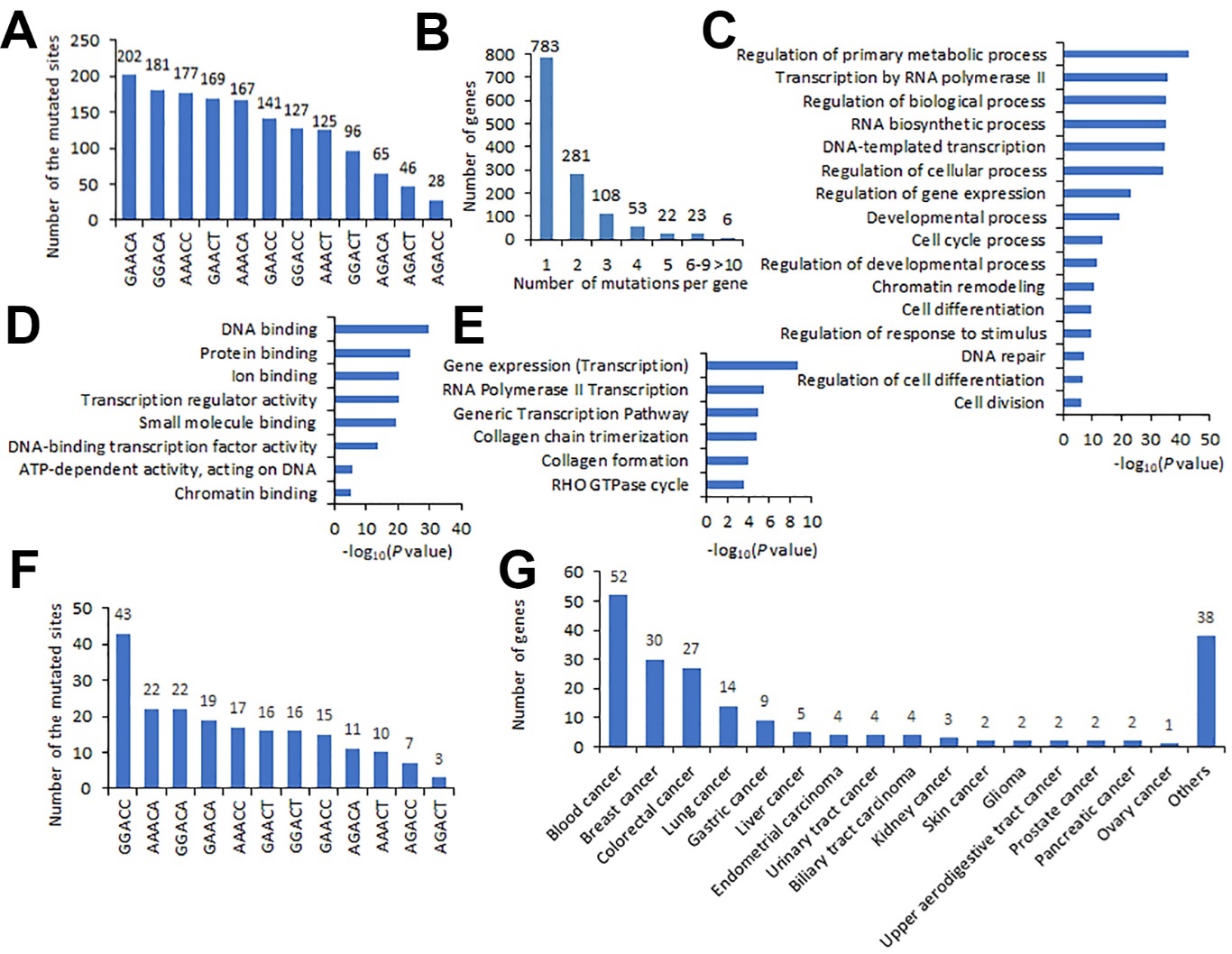


**Figure S1.** Analysis of the synonymous mutations that occurred at the “A” site of the classic m^6^A motifs DRACH with detected m^6^A peaks and have been reported in the literature collected in the PubMed. **(A)** The numbers of synonymous mutations occurring at the “A” site of different m^6^A motifs with detected m^6^A peaks, which have been reported in PubMed literature and are not associated SNPs (i.e., non-SNP-associated). **(B)** The numbers of individual genes that have different numbers of the above non-SNP-associated synonymous mutations. **(C-E)** GO analysis of the genes with the above non-SNP-associated synonymous mutations enriched in the specific Biological Processes (BP; C), Molecular Functions (MF; D), and Reactome pathways (REAC; E). **(F)** The numbers of synonymous mutations occurring at the “A” site of different m^6^A motifs with detected m^6^A peaks, which have been reported in PubMed literature and are also associated with SNPs (i.e., SNP-associated). **(G)** The numbers of the above SNP-associated synonymous mutations detected in different types of human cancers. Others, other types of cancers besides the listed individual types of cancers.


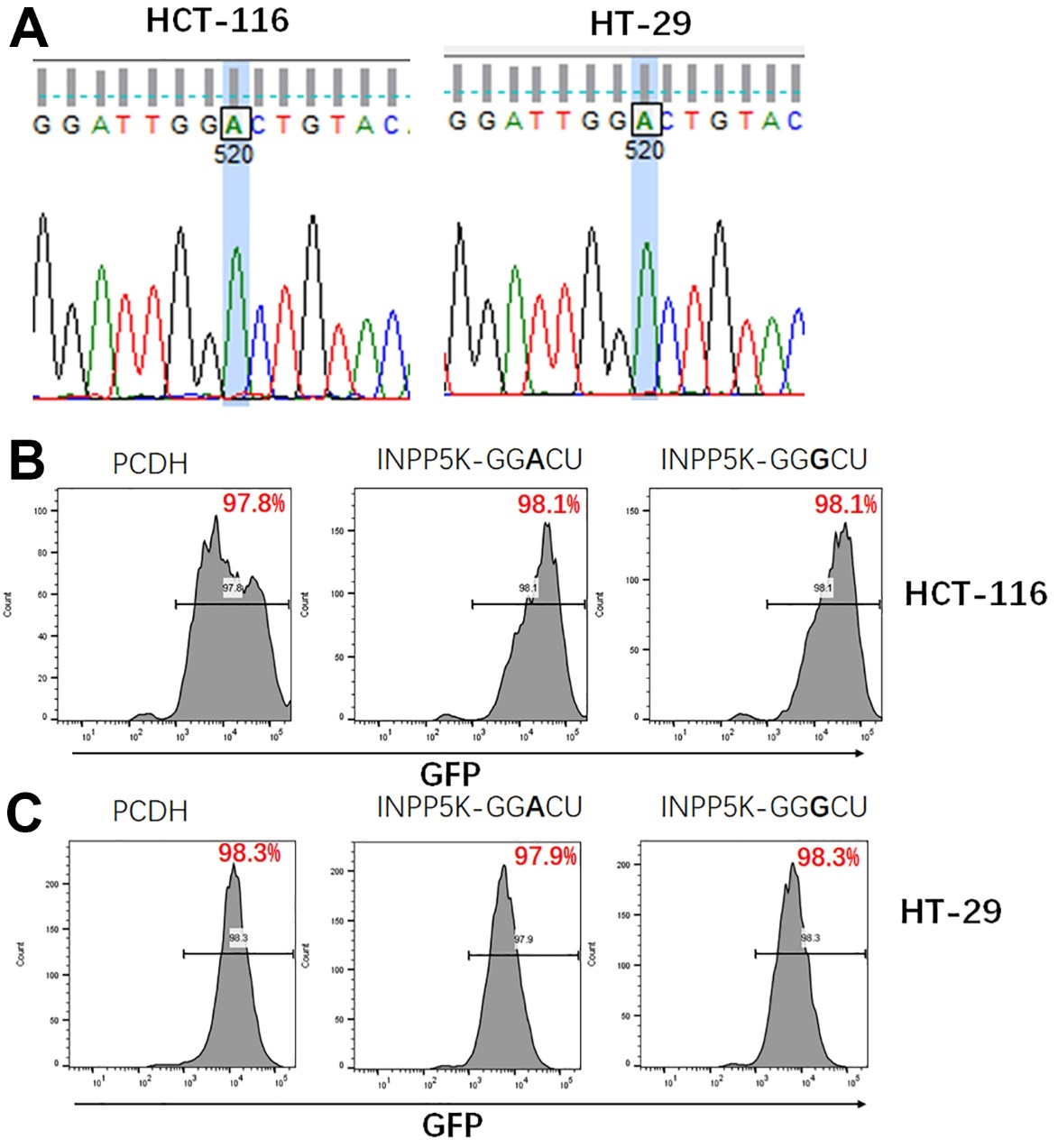


**Figure S2.** Forced expression of wildtype *INPP5K* (INPP5K-GG**A**CU) and mutant *INPP5K* with a synonymous mutation (INPP5K-GG**G**CU) in HCT-116 and HT-29 cells. **(A)** Confirmation of the wildtype endogenous *INPP5K* sequence in HCT-116 and HT-29 cells. **(B and C)** Proportions of transduction positive cells after GFP sorting in HCT-116 (B) and HT-29 (C) cells virally transduced with PCDH empty vector, PCDH-INPP5K-GG**A**CU (wildtype), or PCDH-INPP5K-GG**G**CU (mutant). PCDH vector can also express GFP.
